# Dissolved organic matter quantity and quality response of tropical rainforest headwater rivers to the transition from dry to wet season

**DOI:** 10.1038/s41598-024-53362-z

**Published:** 2024-02-08

**Authors:** S. Norouzi, T. Wagner, A. MacDonald, J. Bischoff, J. Brasche, S. Trojahn, J. Spray, R. Pereira

**Affiliations:** 1 The Lyell Centre, Heriot-Watt University, Edinburgh, UK; 2 British Geological Survey, The Lyell Centre, Edinburgh, UK; 3 Iwokrama International Centre for Rainforest Conservation and Development, Georgetown, Guyana; 4 The James Hutton Institute, Aberdeen, UK

**Keywords:** Carbon cycle, Geochemistry, Hydrology

## Abstract

Dissolved organic matter (DOM) and its composition in aquatic ecosystems is a key indicator of ecosystem function and an important component of the global carbon cycle. Tropical rainforest headwaters play an important role in global carbon cycling. However, there is a large uncertainty on how DOM sources interact during mobilisation and the potential fate of associated carbon and nutrients. Using field techniques to measure dissolved organic carbon (DOC) concentration and composition, changes in DOM source from headwaters to larger downstream rivers were observed. This study shows that the hydrological connectivity, developed during the transition from dry to wet seasons, changes the DOM supply and transport across a tropical river catchment. The observed variability in the DOC-river discharge relationship provides further evidence of the changes in the DOM supply in a small headwater. This novel insight into the seasonal changes of the dynamics of DOM supply to the river helps understanding the mobilization of terrestrial DOM to tropical headwaters and its export from smaller to larger rivers. It also highlights the data gap in the study of smaller headwaters which may account for uncertainty in estimating the terrestrial carbon transported by inland waters.

## Introduction

Inland waters, which include rivers, lakes, and wetlands, are estimated to receive up to 5.7 petagrams of terrestrial carbon per year (Pg C yr^−1^) of which only 0.9 Pg C yr^−1^ reaches the ocean^1–3^. With approximately 0.6 Pg C yr^−1^ buried in sediments, these estimates highlight the role of inland waters as biogeochemical reactors^4^ with the potential to emit ~ 3.9 Pg C yr^−1^ to the atmosphere as CO_2_^1–3^, roughly equal to half the estimated anthropogenic fossil fuel CO_2_ emissions^5^. Tropical river systems such as the Amazon, Congo, Orinoco, and Essequibo^6–9^ are a major component of the global carbon cycle, as they transport and recycle large amounts of carbon and nutrients. The annual flux of dissolved organic carbon (DOC) from tropical rivers to the ocean alone is estimated to account for ~ 59% of the total global riverine flux^10^. Headwaters are estimated to account for 70–80% of the total river network and therefore impact the rate of supply of terrestrially derived organic matter (OM) that has the potential for (a)biotic transformation in the river. The proximity of headwaters to terrestrial OM inputs means that their components directly relate to their immediate surroundings and environmental conditions^11,12^ including soils and vegetation^6,13–15^. The composition of dissolved OM (DOM) therefore reflects either terrestrially-derived (allochthonous) or riverine aquatic (autochthonous) sources^16^. Both can vary significantly both spatially and temporally within a specific study area^17^.

UV–Vis absorbance spectrophotometry has been widely used for characterisation of one prominent portion of DOM, known as coloured DOM (CDOM), due to its practical advantages allowing rapid analysis via in-situ sensors, remote sensing, or in a laboratory^18^. CDOM proxies based on absorption at individual or ranges of wavelengths are commonly used to infer molecular size increases (E_2_:E_3_ and Slope Ratio (S_R_))^19–22^ and aromaticity (SUVA_254_)^23^. As molecular size increases, the E_2_:E_3_ ratio decreases due to stronger light absorption by high-molecular-weight CDOM at longer wavelengths^19^. Samples with low S_R_ are generally of higher molecular weight and have a greater tendency to be allochthonous^22^. However, the contribution of CDOM to the total DOM pool is variable with UV–Vis “invisible” DOM, which has been shown to account for up to ~ 80% of the total DOM pool in lowland tropical headwater river systems^11^. This variability and the potential to miss a large proportion of DOM that may have more affinity to be reworked by photochemical and microbial activity than CDOM highlights the need for further empirical studies in tropical headwater rivers^11^. This is further compounded by difficulties in identifying DOM sources and delivery mechanisms in tropical rivers, such as groundwater^24^ and rain events^11^.

This study is focussed on the Essequibo River catchment in Northern Amazonia because of its high potential for DOM transportation from tropical rainforest through its headwaters to the coastal waters. The study builds upon earlier work by Pereira et al.^7^ to examine the DOC quantity and DOM compositional variability in two headwater streams located in a lowland tropical rainforest in central Guyana, with the aim to elucidate the effect of hydrological seasonality and ground water connectivity on DOM source and supply to the river. The focus is on seasonal responses of DOM to hydrological changes during the transitional limb from the dry to wet season (May and early June 2019).

## Materials and methods

### Study site

**Figure 1:**
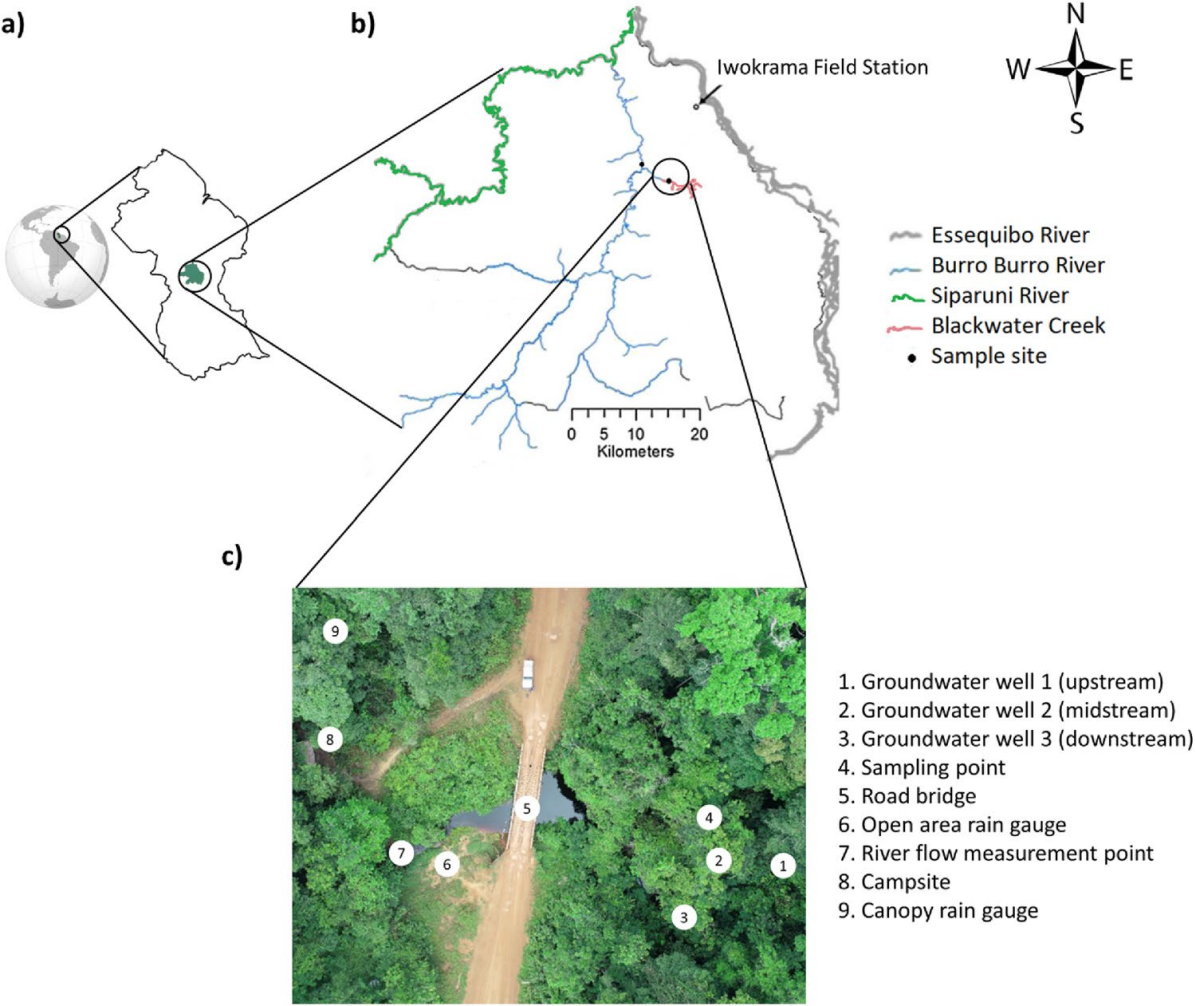
Study site map. The map of (**a**) Guyana, (**b**) the Iwokrama rainforest, located in the Potaro/Siparuni region (modified from ^7^), and (**c**) aerial view of the Blackwater Creek (BC) study site.

The Iwokrama forest is in the heart of the Guiana Shield part of northern Amazonia, in tropical South America, and is bounded by the Essequibo River to the East and the Siparuni River, a tributary of the Essequibo, to the west and north (Fig. 1). The Iwokrama Field Station is located near a transition in the climate regime from the north (coastal) to south (savannah) from two wet seasons (primary: May–July and secondary: December–January) and two dry seasons (primary: centred around October and secondary: centred around March) to one wet season (May–August) and one long dry season (September–March)^7,25^. The Burro-Burro River (BBR), a fifth-order tributary of the Essequibo River, runs through the centre of the forest, and most of its watershed is dominated by forest. The BBR and Blackwater Creek (BC), a second-order headwater of the BBR, were chosen to investigate the temporal and spatial variability of riverine DOM concentration and composition. Both study sites are surrounded by near-pristine forests that contain native species of Mora, Manicole, Crabwood, Trysil, Mixed Greenheart, Black Kakaralli, Wamara, Wallaba, Dakama, Sand Baromalli, and Soft Wallaba trees which are the typical trees in the river BBR catchment.

### Hydrometeorological data collection

Daily rainfall was measured at the Iwokrama field station using a storage rain gauge from January 2016 to December 2019 with the exception of April and May 2019. At the BC study site, rainfall was measured using Adcon RG1 rain gauges equipped with a tipping bucket with 200 cm^2^ orifice area and 0.2 mm resolution from 3rd to 28th May 2019 (no data was recorded in the last 10 days of the study period due to the rain gauge failure) and a storage rain gauge was used to record daily rainfall from 14th May to 7th June 2019.

River stage and groundwater level was measured using sealed Solinst Levelogger Edge pressure transducers recording every 1 min from 3rd May to 7th June 2019. All water levels were corrected for atmospheric pressure changes using a Solinst Barologger installed at BC and BBR study sites. River discharge for BC was calculated from an empirical correlation between the measured discharge and river stage (rating curve) (Refer to Supplementary information, Appendix B). A rating curve is not available for the BBR.

### Sample collection and preservation

To monitor the temporal and spatial variability of DOM, river water samples were collected from BC and BBR from 4th May until 7th June 2019. Groundwater samples were taken at both locations to investigate the potential sources of DOM in river waters. River water was obtained manually during four intensive sampling phases from 4th May 12:00 to 10th May 05:00 (First Phase), 13th May 14:00 to 18th May 05:00 (Second Phase), 21st May 11:00 to 28th May 05:00 (Third Phase) and 31st May 10:00 to 7th June 05:00 (Fourth Phase) from BC. In BBR, river water was collected from 4th May 12:46 to 10th May 11:00 (First Phase), 13th May 17:00 to 18th May 05:00 (Second Phase), 21st May 12:30 to 28th May 05:00 (Third Phase) and 31st May 10:00 to 7th June 10:30 (Fourth Phase). The BBR sampling site is located approximately 50 m downstream from the confluence of the BBR and BC. Grab samples were collected from midstream and the upper 0.3 m of the water column every 6 h at 05:00, 11:00, 17:00 and 23:00 to capture diurnal variation (baseline samples). 95 river water samples were collected from BC. For BBR, 68 river water samples were collected in the first 3 sampling phases and one sample was collected every day for eight days between 08:00 and 10:00 am at BBR during the last sampling phase (8 samples in total). Extra water samples were collected during rainstorm events every 30 min in response to observed hydrological changes such as rainfall and/or increased river discharge with the same collection method as baseline samples (rainstorm event samples: 171 for BC and 116 for BBR).

In addition, 52 groundwater water samples (38 from BC and 14 from the BBR) were collected from 4 fully slotted 4 m wells, 3 in the BC study site (GW1, GW2 and GW3 which were upstream, midstream, and downstream of the river water sampling point, respectively), and 1 in the BBR study site located in parallel to the river sampling site (GW4). Groundwater samples were collected using a PVC bailer allocated to each groundwater well that was pre-rinsed prior to sample collection.

As OM can be transferred from the atmosphere to land and rivers via precipitation (wet deposition)^26^, rainwater itself can be a potential DOM source. Therefore, 12 rainwater samples from an open area and 5 under-canopy rainwater samples were collected at the BC study site. In the BBR study site, 7 under-canopy rainwater samples were collected. Rainwater was collected using a pre-cleaned (acid washed) bucket with samples collected immediately after the rain events.

All water samples were filtered immediately using pre-combusted GF-F filters (450 °C for 8 h) with a nominal pore size of 0.7 µm in the sampling sites. All parts of the filtration equipment were pre-rinsed with sample water before filtration. The filtered water was collected in 60 ml high density polyethylene (HDPE) bottles which were acid-washed (10% HCl) and rinsed with deionised water (18.2 M Ω cm^−1^, carbon-free) prior to use. Samples were stored in the field and transported to mobile laboratory in the Iwokrama river lodge under dark and cold conditions using a portable chiller (4–8 °C).

### Field analysis and mobile laboratory

Water samples were transported daily to the mobile laboratory at the Iwokrama field station where DOC and UV–Vis absorbance (CDOM) analyses were completed within 24 h after collection. In total 247 samples were collected and analysed (171 river water, 24 rainwaters and 52 groundwaters samples). DOC was measured using a Sievers M5310C Portable TOC Analyzer, with attached GE Autosampler with a 0.03 to 50 mg l^−1^ range. Calibration of the analyser was completed prior to the expedition by the manufacturer and sucrose standard solutions (Sigma Aldrich) were prepared on-demand as check standards. DOC results were within the specification of < 1% RSD precision and ± 2% accuracy. UV–Vis absorbance (CDOM) was measured using a Biochrom™ Lightwave II UV–Vis Spectrophotometer from 200 to 800 nm in 1 nm steps using a 1 cm path quartz cuvette pre-rinsed three times with ultrapure water (deionised water). Spectra were blank corrected from 700–800 nm and measurements were reported in Napierian absorbance coefficients *a* (m^−1^) following Hu et al.^27^. The SUVA_254_ was calculated by dividing absorbance coefficients at 254 nm wavelength by the DOC concentration (*a*_254_/DOC)^23^. E_2_:E_3_ is the ratio of absorbance coefficients at 250 nm to 365 nm wavelengths^19–22^. S_R_ is the ratio of *S*_275–295_ to *S*_350–400_ (*S*_275–295_: spectral slope coefficient between 275 and 295 nm wavelengths, *S*_350–400_: spectral slope coefficient between 350 and 400 nm wavelengths)^19–22^.

### Invisible and coloured DOC estimation

To have a better understanding of the compositional changes of DOC in the studied headwaters, the concentration of coloured DOC (CDOC) and UV absorbance invisible (non-absorbing) DOC (iDOC) were estimated based on UV–Vis absorbance model^28^ using measurements made in the mobile field laboratory. This model has been developed using the UV spectra for 1700 surface water samples by Carter et al.^28^ and assumes that DOM consists of three components (A, strongly UV-light absorbing component at 254 nm and B, weakly UV-light absorbing component at 350 nm, and C, non-absorbing DOM often assumed to be constant, 0.80 mg DOC L^−1^)^28^. Therefore, the DOC concentration in a given water sample is as follow:

1 $$\left[DOC\right]=\left[{DOC}_{AB}\right]+[{DOC}_{C}]$$

where [*DOC*_*AB*_] and [*DOC*_*C*_ ] refers to the concentration of light-absorbing and non-absorbing components of the DOC^28^. The relatively low variability of DOC concentration used in the dataset to construct the model and previous work highlighting the presence of UV-invisible DOM in BC^11^ prompts a re-examination of the suitability of a constant non-UV absorbing DOM. Thus, in this study, we consider non-UV absorbing DOC as variable and instead calculate it using the following equation:

2 $$\left[iDOC\right]=\left[DOC\right]-\left[{DOC}_{AB}\right]$$

iDOC has the potential to constitute a notable proportion of the total DOC present with river water and may consist of low molecular weight organic compounds including acids, aldehydes, and ketones^11^.

## Results

### Climate and hydrology

**Figure 2:**
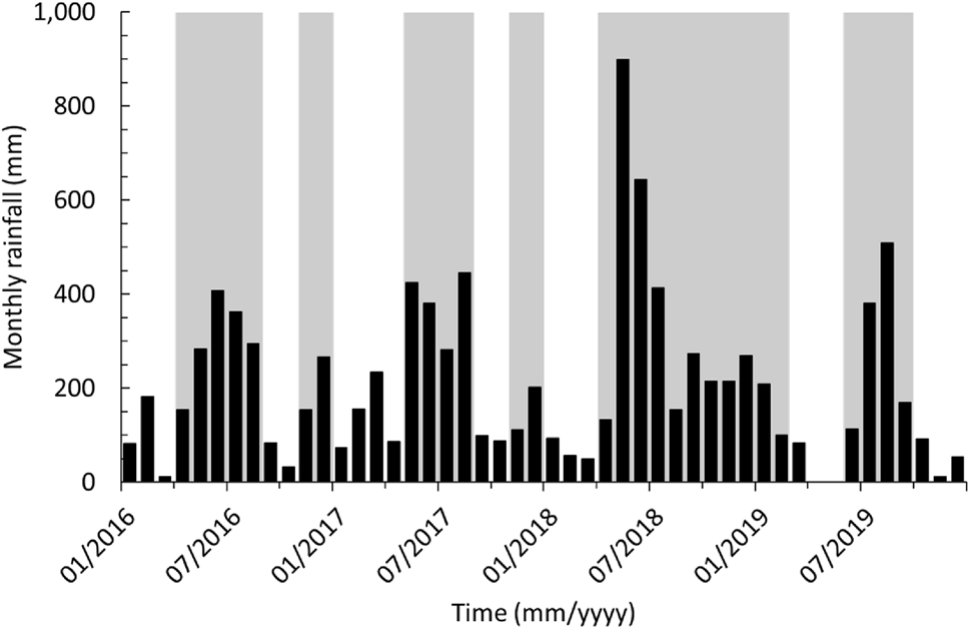
Monthly rainfall data at the Iwokrama Field Station. Measured from January 2016 to December 2019 with wet seasons marked by grey background (No record available for April and May 2019).

Figure 2 shows a near-continuous total monthly rainfall record at the Iwokrama Field Station from January 2016 to December 2019 (no records available for April and May 2019). The annual total rainfall for 2016, 2017, 2018 and 2019 was 2316, 2583, 3414 and 2115 mm, respectively. Following the global climate classification of Peel et al. 2007 and based on the Köppen‐Geiger system for the Guianas (rainfall in the driest month is ≥ 60 mm or < [100 mm – (mean annual rainfall/25)]), 2019 started with a wet season that began in April 2018. The primary dry season started in March 2019 and was followed by a wet season from June to September 2019. Therefore, the study period was conducted at the start of the wet season and can be considered the transitional limb from dry to wet season. The total monthly rainfall in the BC study site for May 2019 was 421 mm.

The river discharge of BC ranged from 0.05 to 5.59 m^3^ s^−1^ with the mean value of 1.42 m^3^ s^−1^ over the study period, which is in the same order of magnitude previously reported for May 2010 and 2011^7^. The river water level ranged from 0.66 to 2.32 m in BC (SD = 0.34).

### DOC concentration spatial and temporal variability

**Figure 3:**
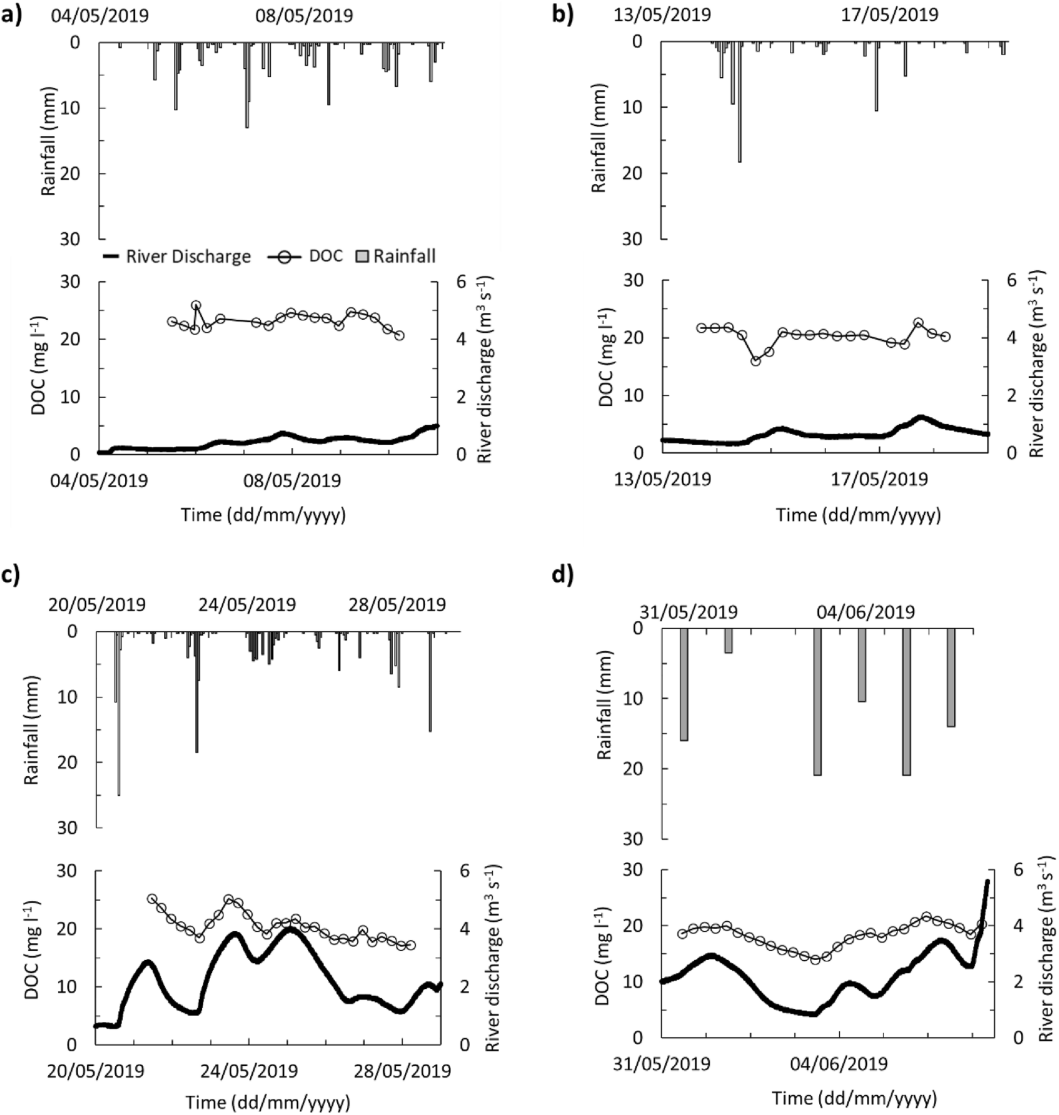
BC DOC concentration (every 6 h), river discharge and rainfall for (**a**) Phase 1, (**b**) Phase 2, (**c**) Phase 3 and (**d**) Phase 4. Hourly rainfall data for phase 1, 2 and 3 (Adcon RG1 rain gauges) and daily rainfall for Phase 4 (storage rain gauge). No hourly rainfall data available in Phase 4 due to rain gauge failure.

Figure 3 shows the hourly rainfall totals, river discharge and DOC concentration for four sampling phases with a temporal resolution of at least 6 h at the BC study site. At BC, the DOC concentration in the base line river water samples ranged from 13.60 to 26.4 mg l^−1^ and generally decreased over the study period. However, at the daily scale, the DOC concentration was variable and changed with river discharge.

**Figure 4:**
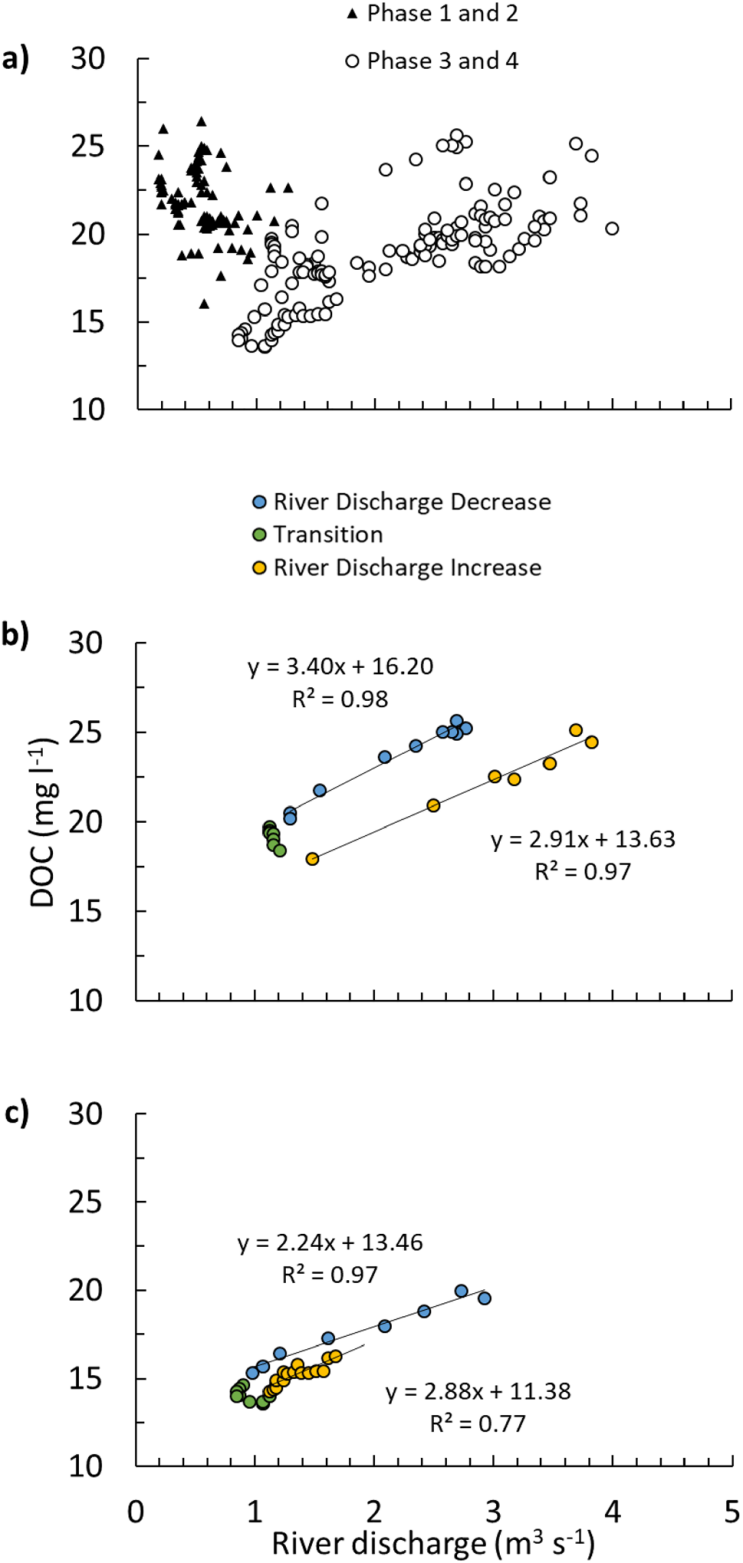
(**a**) A scatterplot of Blackwater Creek (BC) dissolved organic carbon (DOC) concentration and river discharge. (**a**) Phase 1 and 2 (black triangles) and phase 3 and 4 (circles). Plot b and c are shorter periods from phase 3 and 4. (**b**) from 21st May till the end of the 23rd May and (**c**) from 1st till the end of the 3rd June starting with decreasing river discharge (blue), followed by transition from decreasing to increasing river discharge (green) and ending with increasing river discharge (yellow).

In our analysis, a non-parametric linear regression analysis was employed to assess the relationship between DOC concentration and river discharge. In the first phase, the results of the test indicated a weak association between DOC concentration and river discharge (DOC = -0.03 × River discharge + 23.22, *R*^2^ = 0.08, *n* = 57, *p* = 0.554) at BC (Fig. 4 a). In the second phase, the DOC-discharge relationship varied with both increases and decreases in response to two increased river discharge events. In the second half of the study period (Phase 3 and 4), the DOC concentration and river discharge show a linear relationship (DOC = 2.34 × River discharge + 13.94, *R*^2^ = 0.52, *n* = 84, *p* < 0.001) (Fig. 4 a).

Supplementary Table S2 reports the detailed DOC concentration versus river discharge for short time periods (daily scale). The periods were chosen based on the change in the river discharge trend. All of these periods show a weak relationship between DOC and river discharge in the first and second phase (*R*^2^ < 0.50) except the decreasing river discharge on 9th May (*R*^2^ = 0.67, *n* = 11, *p* = 0.002) and increasing river discharge in the 17th May (*R*^2^ = 0.64, *n* = 9, *p* = 0.010). The record from 21st until the end of 23rd May (*n* = 24) captures the decrease in water discharge followed by an increase (Fig. 4b). The relationship between river discharge and DOC starts with a strong linear correlation (*R*^2^ = 0.98, *n* = 10, *p* < 0.001) followed by a transition period at the minimum river discharge and DOC concentration (*n* = 7) followed by a further linear correlation (*R*^2^ = 0.97, *n* = 7, *p* < 0.001) coinciding the increase in the DOC and river discharge. The transition period can be observed in Fig. 4 c showing the record from the 1st to the end of 3rd June capturing another decrease in river discharge followed by a similar increase (*n* = 35).

**Figure 5:**
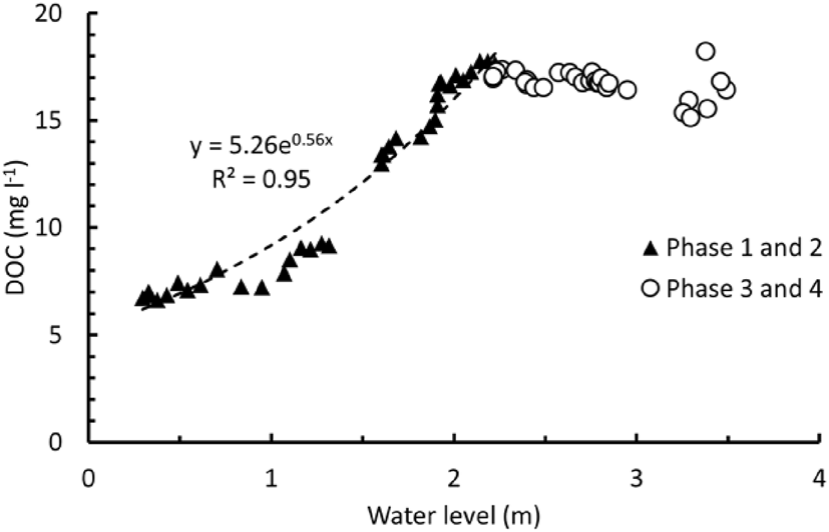
Dissolved organic carbon (DOC) concentration versus river water level at Burro-Burro River (BBR). Phase 1 and 2 (triangles), phase 3 and 4 (circles), and the correlation between DOC and water level in phase 1 and 2 (dashed line).

The DOC concentration in groundwater at BC increased over the study period. GW1 ranged from 12.8 to 21.6 mg l^−1^ (mean value = 19.0 mg l^−1^, *n* = 12), GW2 ranged from 4.0 to 20.5 mg l^−1^ (mean value = 12.7 mg l^−1^, *n* = 11) and GW3 ranged from 7.5 to 25.3 mg l^−1^ (mean value = 12.6 mg l^−1^, *n* = 12) (Supplementary Fig. S1). Notably, GW1 DOC concentration was stable (21.0 ± 0.5 mg l^−1^) when the groundwater level was elevated and constant (1.3 m bgl (below ground level)) from 23rd May to 7th June (Fig. 5).

At the BBR, the DOC concentration in the river water ranged from 6.7 to 18.2 mg l^−1^ during the whole study period. In the first and second phase of the study period, the DOC concentration generally increased and had a wider range from 6.7 to 17.8 mg l^−1^, which could be characterised by a power law relationship with river water level (*R*^2^ = 0.96, *n* = 114, *p* < 0.001; BBR rating curve data not available). In comparison, the DOC concentration of the third and fourth phase of the study period remained relatively stable (14.6 to 18.2 mg l^−1^) while river water level increased. The DOC concentration of GW4 ranged from 8.7 to 14.6 mg l^−1^ and decreased over time (Supplementary Fig. S1).

The rainwater samples from both study sites had DOC concentrations from 1.24 to 4.55 mg l^−1^ for under-canopy rainwater (mean value = 3.3 mg l^−1^, *n* = 12) and from 0.7 to 3 mg l^−1^ in open area rainwaters (mean value = 2.1 mg l^−1^, *n* = 10).

### DOM composition

**Figure 6:**
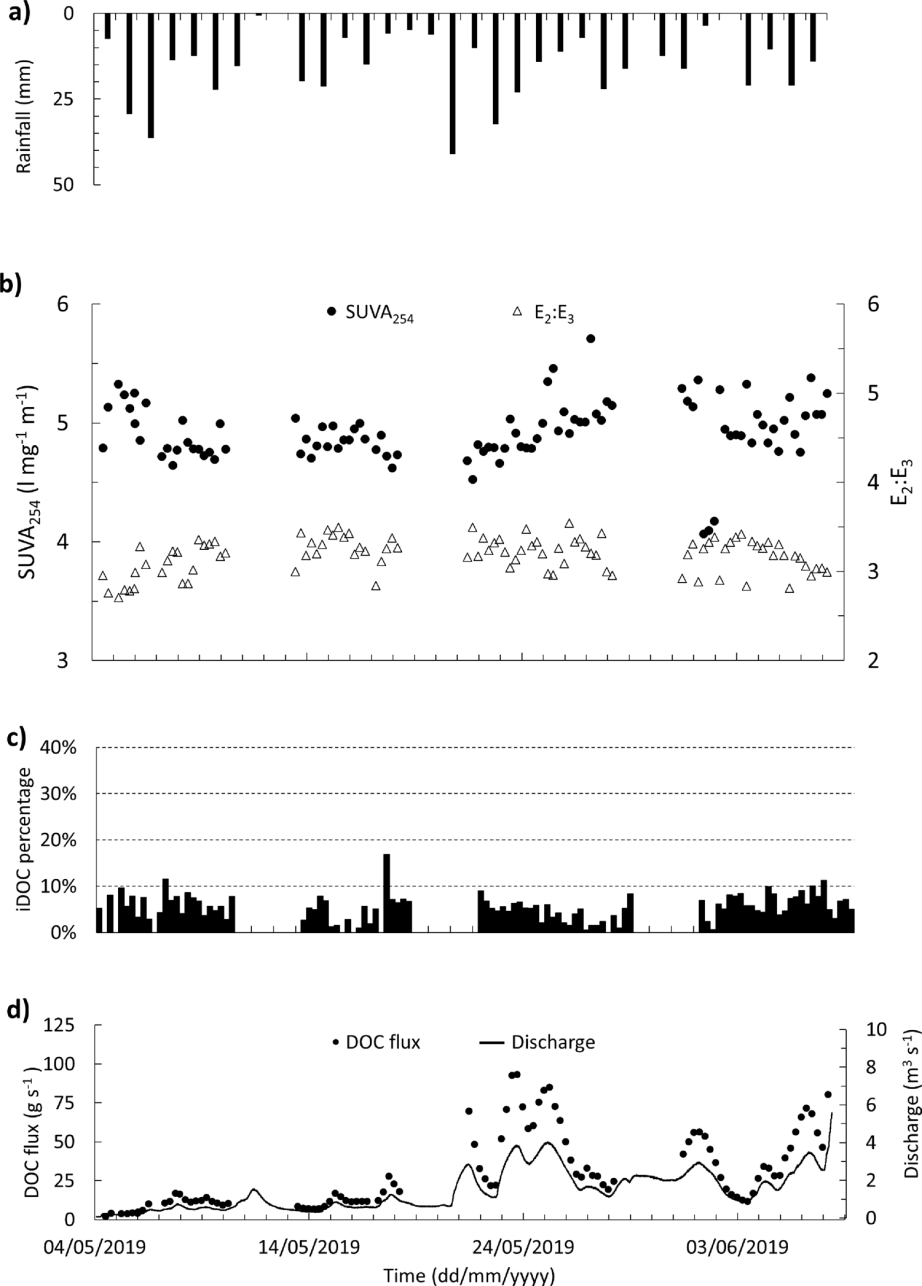
Blackwater Creek (BC) rainfall, discharge, and CDOM. (**a**) daily rainfall, (**b**) SUVA_254_ (black circles) and E_2_:E_3_ coloured dissolved organic matter (CDOM) ratio (triangles), (**c**) calculated invisible dissolved organic carbon (iDOC) every 6 h, and (**d**) river discharge (line) and dissolved organic carbon (DOC) flux every 6 h (black circles).

**Figure 7:**
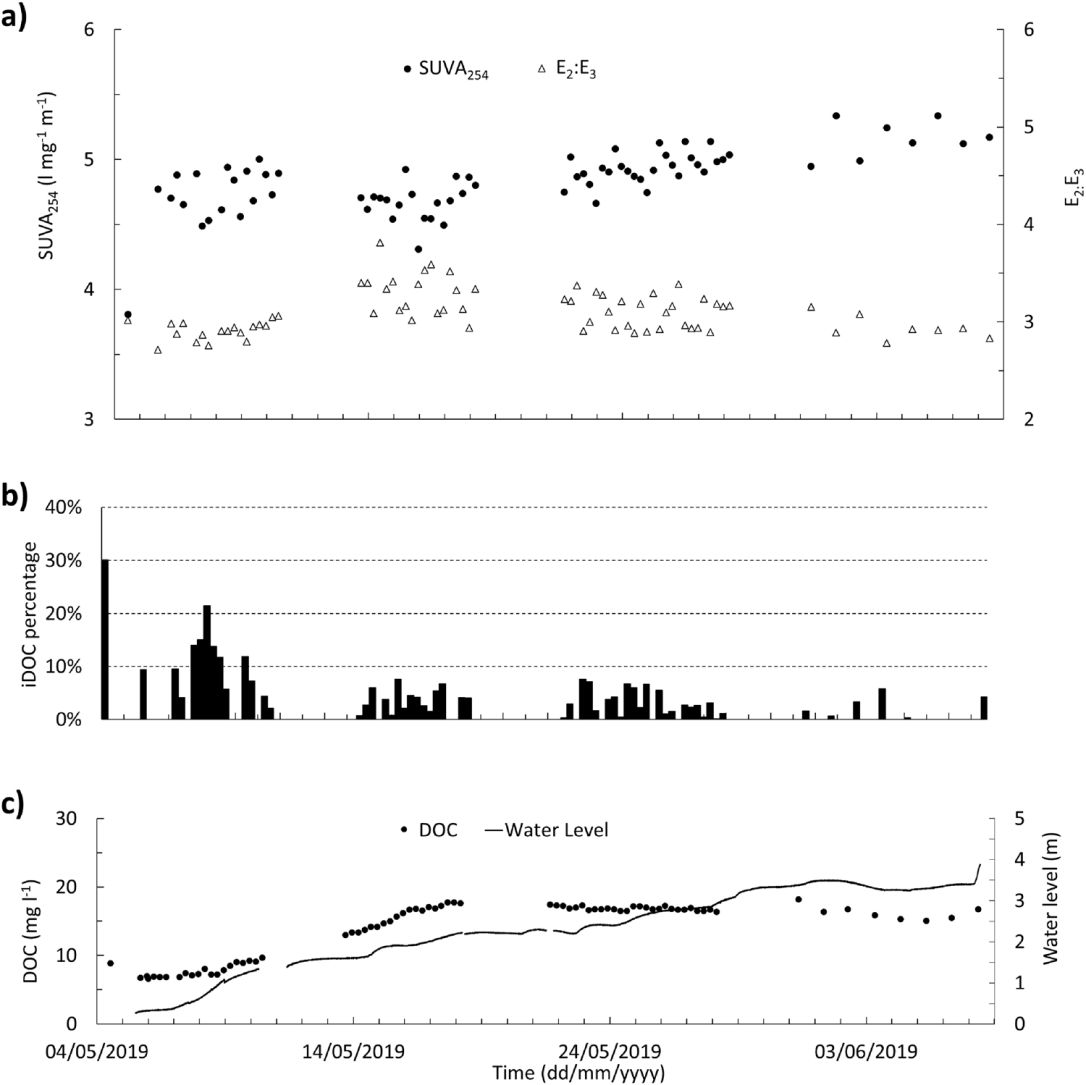
Burro-Burro River (BBR) rainfall, water level, and CDOM. (**a**) SUVA_254_ (black circles) and E_2_:E_3_ ratio (triangles), (**b**) calculated invisible DOC (iDOC) every 6 h, and (**c**) relative water level (line) and dissolved organic carbon (DOC) concentration every 6 h (black circles).

The maximum concentration of iDOC observed in the BBR and BC rivers were 2.7 and 4.6 mg l^−1^, respectively (BBR mean = 0.4 mg l^−1^, BC mean = 1.2 mg l^−1^) with iDOC accounting for up to 17 and 30% of the DOC in BC and BBR, respectively (Figs. 6, 7). Rainwater samples had the highest content of iDOC (93% in the BC and 71% in the BBR). Where negative iDOC values were observed (42 samples), these were adjusted to zero and assumed that *DOC*_*A*_ is more strongly absorbing UV at 254 nm than the originally calibrated model^28^. UV–Vis absorbance model overestimates DOC for 78% of BBR water samples (DOC residual = − 0.4 ± 0.5 mg/l) and underestimates DOC for 75% of BC water samples (DOC residual = + 0.4 ± 0.7 mg/l).

${a}_{254}$ ranges from 102 to 298 m^−1^ in BC and from 74 to 207 m^−1^ in the BBR. Both rivers show a strong relationship between ${a}_{254}$ and DOC concentration ($DOC=0.08*{a}_{254}+0.66$, *R*^*2*^ = 0.89, *n* = 95, *p* < 0.001 at BC and $DOC=0.08*{a}_{254}+0.90$, *R*^2^ = 0.96, *n* = 68, *p* < 0.001 at BBR). SUVA_254_ ranged from 3.8 to 5.3 mg^−1^ m^−1^ in BC and from 4.1 to 5.7 l mg^−1^ m^−1^ in BBR river waters (Supplementary Tables S3, S4). Both rivers showed an increase in SUVA_254_ over the study period, while the BBR displayed generally higher values than BC. In BC groundwater SUVA_254_ ranged from 3.8 to 6.5 l mg^−1^ m^−1^. Rainwater SUVA_254_ ranged from 0.2 to 3.1 and from 3.2 to 5.8 at BC and BBR, respectively (Supplementary Tables S3, S4). Three water samples collected on 1st May at 11:00, 17:00 and 23:00 at BC show lower SUVA_254_ values (4.1, 4.1 and 4.2 l mg^−1^ m^−1^, respectively) in comparison to the rest of the dataset. The E_2_:E_3_ ranged from 2.64 to 3.55 in BC river water, from 1.92 to 3.12 in BC groundwater, from 2.69 to 3.84 in BBR river waters and from 1.83 to 2.76 in BBR groundwater. The SUVA_254_ and E_2_:E_3_ relationship at BC and BBR river water samples show higher SUVA_254_ values have lower E_2_:E_3_ values. The SUVA_254_ and E_2_:E_3_ relationship in groundwater samples is stronger (*R*^*2*^ = 0.71, *n* = 35, *p* < 0.001) than river water samples (*R*^2^ = 0.35, *n* = 95, *p* < 0.001) at BC. The under canopy rainwater samples also shows a decrease of E_2_:E_3_ as SUVA_254_ increases (*R*^2^ = 0.73, *n* = 5, *p* = 0.16). For rainwater samples, E_2_:E_3_ increases as SUVA_254_ increases (*R*^2^ = 0.48, *n* = 12, *p* = 0.11). In the BBR the SUVA_254_ and E_2_:E_3_ relationship in groundwater is stronger (*R*^2^ = 0.76, *n* = 15, *p* = 0.001) than river water samples (*R*^2^ = 0.13, *n* = 68, *p* = 0.330). The under-canopy rainwater samples at BBR show a similarly strong relationship between SUVA_254_ and E_2_:E_3_ (*R*^2^ = 0.92, *n* = 7, *p* = 0.003). The S_R_ ranged from 0.61 to 0.85 in BC river water, from 0.65 to 1.13 in BC groundwater, from 0.46 to 0.92 in BBR river water and from 0.79 to 1.00 in the BBR groundwater (Supplementary Tables S3, S4).

### Riverine fluxes

DOC, iDOC and CDOC flux was estimated by multiplying the concentration and river discharge. The estimated DOC flux of BC was 2.0 g s^−1^ (0.5 g s^−1^ iDOC) at the start of the study period and increased to 119.0 g s^−1^ (9.2 g s^−1^ iDOC) at the end of the study period. BC is estimated to have transported 13.1 tonnes DOC (0.8 tonnes iDOC) during Phase 1 and 2 and 83.4 tonnes DOC (4.7 tonnes iDOC) during Phase 3 and 4. Table 1 provides the details of the DOC, iDOC, and CDOC flux in each phase of the study period.

**Table 1:** The DOC, iDOC, and CDOC flux of BC in the transition from dry to wet season.

	DOC (tonne/day)	iDOC (tonne/day)	CDOC (tonne/day)
Phase 1	0.83	0.06	0.77
Phase 2	1.27	0.09	1.18
Phase 3	4.13	0.40	3.73
Phase 4	3.53	0.29	3.24

## Discussion

The dry and wet seasons in the Iwokrama rainforest from January 2016 to December 2019 have been comparable with seasonal patterns reported from January 2009 to December 2011 and more widely using climate reanalysis approaches (Fig. 2)^7,25^. The study period (May and June 2019) represents the transitional limb from dry to wet season in the Iwokrama rainforest. During this transition, a wetting up phase where increases in rainfall are reflected in increases in groundwater levels and river discharge were observed, both at the smaller BC and larger BBR scales (Figs. 6d, 7c). However, there is a distinct change in the relationship between the drainage of the catchment and flux of DOC. Phase 1 and 2 reflect drier conditions that likely result in more localised hydrological flow paths, whereas the Phase 3 and 4 hydrological characteristics suggest the catchment was more widely connected with more distal sources and shorter residence times in BC^29,30^.

As BBR water is sourced from smaller headwaters including BC, the hydrological behaviour of smaller headwaters is reflected in the bigger downstream river with the first and second halves of the study period also distinct in the BBR. The water level is less variable in the first half of the study period in BC and BBR. In the second half, the water level is more variable in BC which is reflected in the BBR water level by simultaneous local maxima and minima (for example minima presented in Fig. 4b, c). At the smaller headwater scale, a strong linear correlation (BC water level = 1.00 × GW3 water level + 2.88, *R*^2^ = 0.99, *n* = 50,200, *p* < 0.0001 and BC water level = 0.98 × GW2 water level + 0.53, *R*^2^ = 0.97, n = 50,200, *p* < 0.0001) between river level and mid- and downstream groundwater levels indicates that groundwater and surface waters are well connected during our study period.

The DOC concentration in the BBR and BC is comparable to other studied tropical rivers and shows its high potential for transportation of OM in the Iwokrama rainforest^31–35^. The low S_R_ values (0.46–0.92) observed in this study suggest that CDOM had a higher molecular weight and a greater tendency to be allochthonous. The river water S_R_ values are comparable to data reported for Epulu River, Democratic Republic of Congo^31^ (0.80–1.07) and tropical rivers (Orinoco, Cuyuni, and Paragua) of the Guyana Shield, Venezuela (0.71–0.94)^34^. The similar range of S_R_ in different tropical river basins could imply the similarity of the OM source or transformation processes among these tropical rivers. SUVA_254_, which relates to DOM aromaticity, is consistent with S_R_ suggesting the Iwokrama river waters are strongly sourced from allochthonous sources. However, observed SUVA_254_ (3.8 to 5.7 l mg^−1^ m^−1^) demonstrate a wider range than tropical rainforest streams of Costa Rica (Epulu River (3.08–3.57 mg^−1^ m^−1^)), which may reflect a wider variety of DOM sources entering the river as suggested for the Arboleda and Taconazo streams (SUVA_254_ 0.31–5.36 mg^−1^ m^−1^)^24^, or instream transformations that undergo biotic or abiotic transformation during transport resulting in a different range of organic compounds in the water^36^. The SUVA_254_ values are consistent with groundwater dominated transport of DOM to the river; however, the E_2_:E_3_ ratio, which is inversely correlated with the molecular size, is lower in groundwater than in the BC river water (~ 23%). This could be indicative of biochemical degradation where higher molecular weight DOM would appear more recalcitrant, or indicative of long residence times and/or more distant DOM sources in the groundwater^37–40^.

The elevated SUVA_254_ value observed in the rainwater samples may arise from the contact of rainwater with vegetation, leading to the acquisition of organic compounds (i.e. throughfall). Alternatively, the presence of inorganic constituents may contribute to a higher SUVA_254_ signal. The relationship between SUVA_254_ and E_2_:E_3_ in the groundwater shows that generally, the aromatic components in the groundwater samples have higher molecular size and therefore the higher abundance increases the SUVA_254_ and decreases the E_2_:E_3_. The relationship between SUVA_254_ and E_2_:E_3_ in river water is stronger than in the collected rainwater. This indicates that the DOM sourced from the groundwater to river water plays a major role in controlling the river water DOM composition. The river DOM received from precipitation likely adds stochasticity to the SUVA_254_ and E_2_:E_3_ relationship either through dilution of the river water or addition of DOM.

The observed strong relationship between groundwater and BC water level suggests that shallow groundwater (0.5 m and deeper) is highly connected to the river in this headwater catchment. While DOM from surface soils (< 0.3 m below ground level (bgl)) are a potent and relevant source of riverine DOM which could be mobilized by rainfall^11^, based on observations of DOM composition from this study, groundwater flow paths are also important. In the main stem of the BBR, a clear link between groundwater levels and rising river levels could not be established indicating a weaker coupling between groundwater and surface water on this higher order stream. As would be expected from a higher-order river, the water level continuously and gradually raised in the BBR during the first half of the study period with much less variability in comparison to BC. We note that the observed hydrological response to seasonal changes of the region was different for smaller and larger scale headwaters emphasising the importance of studying a set or continuum of smaller headwaters to support a comprehensive interpretation of the hydrological responses of the catchment based on the larger rivers.

At the lower-order “headwater” scale, the river contained the highest DOC concentration compared to meteoric and groundwaters. Despite the limitations of the UV–Vis absorbance model in accurately estimating DOC concentrations within the highly variable headwaters under study, our dataset offers the potential to examine changing sources of DOC to the river water. The addition of meteoric water to the river water, with its low DOC (almost 15% of river water DOC concentration) and high iDOC percentage (around 15 times more than in river waters), may result in the dilution of DOC concentration at the BC. However, the localised nature of rain events in the study catchment makes estimates of rainfall over the wider catchment challenging. Nevertheless, despite the dilution of the river water DOC, the overall flux of the DOC, iDOC and coloured DOC increased as more water entered the system as part of the transition from the dry to wet season. While rainwater was not likely the dominant DOM source, high potential to mobilize DOM from changing the surface/subsurface dynamics of supply to the river water is expected^30,41,42^. Either way, where DOC concentrations decreased while discharge increased in the dry transition period (Phase 1 and 2) it likely suggests a shunting (rapid transport downstream) of DOC poor water in the catchment ^43^ via land or directly through meteoric supply (Fig. 3a, b).

Interpretation of the chemodynamic behaviour of these headwaters helps the better understanding of the response of the river catchment to the seasonal changes^44^. The strong linear relationship between DOC concentration and river discharge over short time periods (hours to days) in the second half of the study period in BC (Fig. 4b, c) provides further evidence on the changes of the source and amount of available DOM on a daily scale (Supplementary Table S2). The rise in the river water level will increase the surrounding area washed by the river water and therefore the amount of DOC entering the river. As the water level fluctuates at a daily scale, the areal spread and depth of horizons of the soil washed by the river water changes during the study period and part of them are washed more frequently than the others. This highly dynamic process would result in the different relationships observed between DOC and river discharge at the daily scale. Interestingly, there were periods in the wetter stage where there was no relationship between river discharge and DOC concentration despite the strong relationships before and after (Fig. 4b, c). These periods do not reflect insensitivity of the DOC concentration to river discharge (chemostatic behaviour)^44^ and could be considered the transition between rising and falling river discharge, recharge and drainage of surrounding soils, changing sources of the DOM and water to the river, or the time lag between the rise/fall of the water level and access to the DOM source. The close interaction between the headwaters and surrounding soil horizons, emphasizes the importance of further study on the OM sourced from soil and its impact on the composition and quantity of the DOM transported into the river water. The high temporal resolution of our BC data demonstrates the importance of relating daily scale hydrological events into the seasonal context as notable changes were observed in the DOC-discharge and DOC-water level relationship to examine mobilisation, subsequent processing of organic compounds, and determination of carbon fluxes (Figs. 4, 5).

In the larger BBR, the DOC-river water level relationship is notably different to the BC with DOC concentrations corresponding to the overall BBR water level trend in the first half of the study period but more stable in the second half (Fig. 5), suggesting a fully hydrologically connected catchment with constant DOC supply and a potential similar source. This is consistent with observations from other Amazonian river‐wetland systems at the seasonal scale^45^. Based on these observations, the first half of the study period, coinciding with the dry season, could be capturing multiple localised DOM sources to the BC which explains the weaker relationship between river discharge and DOC concentration. At BBR, DOC increases in response to the water level rise in the drier period as headwaters such as BC bring the mobilized DOC from different sources to the bigger river. BBR reflects the wider catchment response to increase in precipitation which adds more water to the streams and soil. As the river discharge is a non-linear function of the river water level (i.e. a rating curve), the DOC and river water level of the BBR shows a non-linear correlation as illustrated by an exponential relationship ($DOC\, concentration = 5.26e^{0.56 \times river \,discharge}$) (Fig. 5). In the second half of the study period, which coincides with the beginning of the wet season, we observe more pronounced relationship between DOC concentration and river discharge. Clear patterns emerge in the DOC during periods of rising, falling, and transitioning river discharge, indicating a noticeable hysteresis effect in the BC^30^. At the BBR, the later study period shows that the catchment is wet and continuously sourced from its headwaters and other sources such as precipitation and groundwater. As groundwater level rises at the later study period, it can source DOC from shallower sections of the soil, too. The slight decrease in the DOC concentration could be a result of the horizons of the soil being regularly washed by headwaters of BBR (such as BC). The higher iDOC content of the bigger river emphasizes the role of multiple sources other than BC-like rivers in supplying DOM to the BBR at dry periods.

The spatial and temporal variability in the DOC-river discharge relationship increases the uncertainty in the estimation of the overall load of DOC transported by the river catchment. We demonstrate the response of smaller headwaters to the short and long term seasonal precipitation changes in bigger rivers. However, higher-order tributaries have the potential to disguise the riverine DOM signals of the lower-order tributaries. This study further shows that lower-order tributaries introduce a higher variability and likelihood of greater uncertainty in the estimation of the DOM flux than the higher-order tributary. Therefore, the interpretation of the impact of seasonal changes on the transportation and flux of DOM from terrestrial ecosystems through tropical inland waters must be based on investigating both smaller and larger headwaters.

## Conclusions

In the transition from dry to the wet season, the interplay between precipitation and the catchment hydrological response is critical to constrain the dynamics of the DOM supply to the river. The dynamics of the DOM supply to the river water in the dry season is likely associated with localised hydrological flow paths and a wider variety of local DOM sources compared to the wet season. Such conditions are characterized by widely hydrologically connected flow paths and more distal DOM sources. The highly dynamic process of water level fluctuation and DOC mobilization and transport to the river introduces variability in the river discharge and DOC relationship in daily scale in the smaller headwaters. Despite the close connection of the smaller headwaters of tropical rainforests to terrestrial ecosystems where there is faster response to hydrological changes and higher DOC concentration in comparison to the bigger rivers, the magnitude of their role in transporting DOM compared to the whole catchment remains elusive. The importance of relating daily scale hydrological events into the seasonal context to constrain changes in DOM sources and fluxes from headwaters to the ocean is a next critical step in improving our estimates and reducing associated uncertainties of large tropical rivers.

## Data availability

The datasets used and/or analysed during the current study available from the corresponding author on reasonable request.

### Supplementary Information

Supplementary Information.
