# Supplementary material for: Dissolved organic matter quantity and quality response of tropical rainforest headwater rivers to the transition from dry to wet season
Source: Sci Rep. 2024 Feb 8;14:3270. doi: 10.1038/s41598-024-53362-z (PMC10853192; doi:10.1038/s41598-024-53362-z)
Supplement: Supplementary file 1 — Supplementary Information. [file 41598_2024_53362_MOESM1_ESM.docx]

# **Appendix A: Rainfall Data**

**Supplementary Table S1:** The rainfall (mm) data collected by storage rain gauge from 14^th^ May 2019 to 6^th^ June 2019 and Adcon RG1 rain gauge from 3^rd^ to 28^th^ May 2019 in the BC study site.

| Storage rain gauge | | Adcon RG1 rain gauge | |
| --- | --- | --- | --- |
| Date | **Rain (mm)** | **Date** | **Rain (mm)** |
| 14/05/2019 | 40.75 | 03/05/2019 | 0.75 |
| 15/05/2019 | 2.25 | 04/05/2019 | 7.25 |
| 16/05/2019 | 7.75 | 05/05/2019 | 29.25 |
| 17/05/2019 | 17.5 | 06/05/2019 | 36.25 |
| 18/05/2019 | 2.25 | 07/05/2019 | 13.50 |
| 19/05/2019 | 8.75 | 08/05/2019 | 12.25 |
| 20/05/2019 | 40.00 | 09/05/2019 | 22.25 |
| 21/05/2019 | 4.00 | 10/05/2019 | 15.25 |
| 22/05/2019 | 38.75 | 11/05/2019 | 0.50 |
| 23/05/2019 | 0.50 | 12/05/2019 | 0.00 |
| 24/05/2019 | 36.75 | 13/05/2019 | 19.75 |
| 25/05/2019 | 5.00 | 14/05/2019 | 21.25 |
| 26/05/2019 | 8.50 | 15/05/2019 | 7.00 |
| 27/05/2019 | 12.50 | 16/05/2019 | 14.75 |
| 28/05/2019 | 29.75 | 17/05/2019 | 5.75 |
| 29/05/2019 | 0.50 | 18/05/2019 | 4.75 |
| 30/05/2019 | 12.25 | 19/05/2019 | 6.00 |
| 31/05/2019 | 16.00 | 20/05/2019 | 41.00 |
| 01/06/2019 | 3.48 | 21/05/2019 | 10.00 |
| 02/06/2019 | 0.00 | 22/05/2019 | 32.25 |
| 03/06/2019 | 20.91 | 23/05/2019 | 23.00 |
| 04/06/2019 | 10.45 | 24/05/2019 | 14.00 |
| 05/06/2019 | 20.91 | 25/05/2019 | 11.00 |
| 06/06/2019 | 13.94 | 26/05/2019 | 7.00 |
|  |  | 27/05/2019 | 22.00 |
|  |  | 28/05/2019 | 16.00 |

# **Appendix B: River Discharge and DOC Flux Calculations**

The BC river discharge for the whole study period was calculated from an empirical correlation between the measured discharge and river stage (rating curve) which is as follows:

| $Q=2.309 {(S-0.57)}^{1.575}$ | (1) |
| --- | --- |

where Q is river discharge (m^3^ s^-1^) and S is the river stage (m). The riverine DOC flux for BC was calculated using the following equation:

| $F=Q C$ | (2) |
| --- | --- |

where C is the DOC concentration (mg l^-1^) and F is the DOC Flux (g s^-1^). As the DOC concentration responds linearly to river discharge in the second half of the study period (Phase 3 and 4), the riverine DOC flux had a quadratic relationship with the river discharge (shown in Table S2) which is as follows:

| $F=aQ^{2}+bQ$ | (3) |
| --- | --- |

where a and b are arbitrary constants.

**Supplementary Table S2:** The dissolved organic carbon (DOC) concentration (mg l^-1^) and discharge (Q (m^3^ s^-1^) relationship for periods of steady river discharge trend (falling (F)/raising (R)/constant (C) water discharge trend at BC.

| Relationship | *R^2^* | Date | Number of Samples | P-Value | F/R/C |
| --- | --- | --- | --- | --- | --- |
| $\boldsymbol{DOC}\mathbf{=-34.86}\boldsymbol{Q}\mathbf{+29.64}$ | 0.48 | 5^th^ May | 10 | 0.027 | C |
| $\boldsymbol{DOC}\mathbf{=-6.92}\boldsymbol{Q}\mathbf{+25.73}$ | 0.15 | 6^th^ May | 4 | 0.630 | R |
| $\boldsymbol{DOC}\mathbf{=-0.13}\boldsymbol{Q}\mathbf{+23.46}$ | 6E-5 | 7^th^ May | 7 | 0.990 | R |
| $\boldsymbol{DOC}\mathbf{=2..01}\boldsymbol{Q}\mathbf{+22.68}$ | 0.05 | 8^th^ May | 10 | 0.540 | C |
| $\boldsymbol{DOC}\mathbf{=18.34+14.70}$ | 0.67 | 9^th^ May | 11 | 0.002 | F |
| $\boldsymbol{DOC}\mathbf{=3.58}\boldsymbol{Q}\mathbf{+20.23}$ | 0.04 | 13^th^ till mid-14^th^ May | 15 | 0.475 | C |
| $\boldsymbol{DOC}\mathbf{=-5.44}\boldsymbol{Q}\mathbf{+20.88}$ | 0.28 | Second half of 14^th^ May | 5 | 0.358 | C |
| $\boldsymbol{DOC}\mathbf{=0.27}\boldsymbol{Q}\mathbf{+20.51}$ | 0.01 | 15^th^ May | 12 | 0.757 | F |
| $\boldsymbol{DOC}\mathbf{=1.85}\boldsymbol{Q}\mathbf{+19.59}$ | 0.02 | 16^th^ May | 9 | 0.719 | C |
| $\boldsymbol{DOC}\mathbf{=7.02}\boldsymbol{Q}\mathbf{+13.4}$ | 0.64 | 17^th^ May | 9 | 0.010 | R |
| $\boldsymbol{DOC}\mathbf{= 3.57}\boldsymbol{Q}\mathbf{+}\mathbf{15.77}$ | 0.99 | 21^st^ till mid-22^nd^ May | 13 | <0.001 | F |
| $\boldsymbol{DOC}\mathbf{=2.20}\boldsymbol{Q}\mathbf{+15.98}$ | 0.93 | Mid-22^nd^ till end of 23^rd^ May | 11 | <0.001 | R |
| $\boldsymbol{DOC}\mathbf{=3.81}\boldsymbol{Q}\mathbf{+7.24}$ | 0.82 | 24^th^ May | 12 | <0.001 | R |
| $\boldsymbol{DOC}\mathbf{=1.29}\boldsymbol{Q}\mathbf{+16.42}$ | 0.79 | 25^th^ till the end of 27^th^ May | 14 | <0.001 | F |
| $\boldsymbol{DOC}\mathbf{=2.20}\boldsymbol{Q}\mathbf{+13.98}$ | 0.61 | 31^st^ May | 18 | <0.001 | R |
| $\boldsymbol{DOC}\mathbf{=}\mathbf{2.71}\boldsymbol{Q}\mathbf{+12.33}$ | 0.94 | 1^st^ till mid-3^rd^ June | 13 | <0.001 | F |
| $\boldsymbol{DOC}\mathbf{=}\mathbf{3.65}\boldsymbol{Q}\mathbf{+10.27}$ | 0.82 | Second half of 3^rd^ June | 20 | <0.001 | R |
| $\boldsymbol{DOC}\mathbf{=-0.008+17.92}$ | 1E-5 | 4^th^ June | 11 | 0.998 | F and R |
| $\boldsymbol{DOC}\mathbf{=2.48}\boldsymbol{Q}\mathbf{+12.95}$ | 0.56 | 5^th^ June | 16 | <0.001 | R |
| $\mathbf{DOC=2.43}\boldsymbol{Q}\mathbf{+12.49}$ | 0.97 | 6^th^ June | 4 | 0.020 | F |

# **Appendix C: Groundwater Wells**


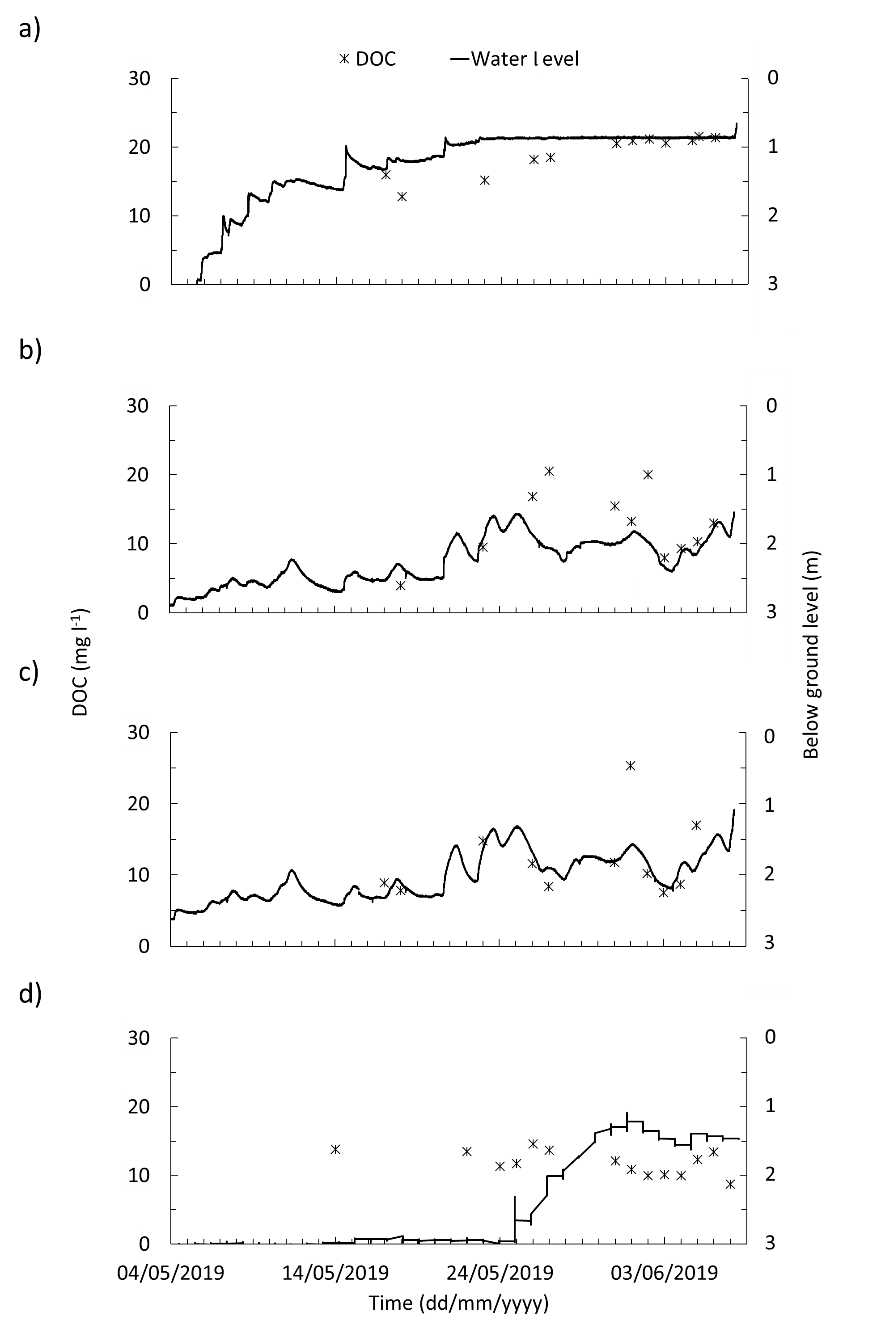


**Supplementary Figure S1:** Dissolved organic carbon (DOC) concentration and water level of a) upstream, b) midstream and c) downstream groundwater wells at Blackwater Creek (BC) and d) groundwater well at Burro-Burro River (BBR).

# **Appendix D: DOC Concentration and CDOM Proxies**

**Supplementary Table S3:** The dissolved organic carbon (DOC) concentration (mg l^-1^), SUVA_254_ (l mg^-1^ m^-1^), E_2_:E_3_, S_R_ and invisible DOC (iDOC) percentage of total DOC mean, standard error and range for BC river, rain and groundwater.

|  | Mean +/-standard error  (min - max) | | | | |
| --- | --- | --- | --- | --- | --- |
|  | **DOC** | **SUVA_254_** | **E_2_:E_3_** | **S_R_** | **iDOC** |
|  | **mg l^-1^** | **l mg^-1^ m^-1^** |  |  | **% of total DOC** |
| River | 20.23 +/-0.14  (13.60 - 26.41) | 4.9 +/- 0.01 (4.1 - 5.7) | 3.16 +/-0.01 (2.64 - 3.55) | 0.69 +/-0.003 (0.61 - 0.85) | 6.04 +/-0.18  (0 - 17.21) |
| Groundwater - upper reach | 18.99 +/-0.84  (12.8 0- 21.60) | 4.6 +/-0.001 (4.1 - 4.9) | 2.88 +/-0.06 (2.49 - 3.12) | 0.73 +/-0.013 (0.65 - 0.80) | 16.75 +/-11.23 (0 - 100) |
| Groundwater - lower reach | 12.64 +/-1.60  (7.50 - 25.30) | 4.6 +/-0.001 (3.9 - 5.3) | 2.77 +/-0.06 (2.27 - 3.04) | 0.77 +/-0.02 (0.71 - 0.91) | 30.95 +/-12.72 (0 - 100) |
| Rainwater – under-canopy | 3.33 +/-0.60  (1.24 - 4.54) | 2.3 +/-0.4  (0.7 - 3.1) | 4.41 +/-1.06 (2.28 - 8.40) | 0.71 +/-0.12 (0.56 - 0.94) | 93.16 +/-6.84 (65.80 -100) |
| Rainwater –  open | 2.09 +/-0.19  (0.72 - 2.99) | 2.5 +/-0.6  (0.2 - 8.8) | 2.37 +/-0.12 (1.77 - 3.04) | 0.60 +/- 0.06 (0.28 - 0.77) | 92.39 +/-7.61 (8.72 - 100) |

**Supplementary Table S4:** The dissolved organic carbon (DOC) concentration (mg l^-1^), SUVA_254_ (l mg^-1^ m^-1^), E_2_:E_3_, S_R_ and invisible DOC (iDOC) percentage of total DOC mean, standard error and range for BBR river, rain and groundwater.

|  | Mean +/-standard error  (min - max) | | | | |
| --- | --- | --- | --- | --- | --- |
|  | **DOC** | **SUVA_254_** | **E_2_:E_3_** | **S_R_** | **iDOC** |
|  | **mg l^-1^** | **l mg^-1^ m^-1^** |  |  | **% of total DOC** |
| River | 14.57 +/-0.26 (6.66 - 18.22) | 4.9 +/-0.02 (3.8 - 5.6) | 3.14 +/-0.02 (2.69 - 3.84) | 0.72 +/-0.005 (0.46 - 0.92) | 3.55 +/-0.33  (0 - 30.14) |
| Groundwater - upper reach | 11.86 +/-0.48 (8.70 - 14.60) | 7.4 +/-0.9  (4.8 - 15.7) | 2.42 +/-0.08 (1.83 -2.77) | 0.92 +/-0.02 (0.79 - 1.00) | 41.63 +/-12.17 (0 - 100) |
| Rainwater – under-canopy | 8.09 +/-1.94 (2.82 - 16.00) | 4.0 +/-0.3  (3.2 - 5.7) | 2.68 +/-0.21 (1.51 - 3.19) | 0.84 +/-0.07 (0.48 - 1.00) | 71.43 +/-18.44 (0 - 100) |
